# Artefacts in contrast enhanced digital mammography: how can they affect diagnostic image quality and confuse clinical diagnosis?

**DOI:** 10.1186/s13244-019-0811-x

**Published:** 2020-02-07

**Authors:** Jacopo Nori, Maninderpal Kaur Gill, Chiara Vignoli, Giulia Bicchierai, Diego De Benedetto, Federica Di Naro, Ermanno Vanzi, Cecilia Boeri, Vittorio Miele

**Affiliations:** 1 Diagnostic Senology Unit, Azienda Ospedaliero-Universitaria Careggi, Florence, Italy; 2 Department of Radiology, Tengku Ampuan Rahimah Hospital, Klang, Malaysia; 3 Department of Radiology, Azienda Ospedaliero-Universitaria Careggi, Florence, Italy

**Keywords:** Breast cancer, Artifacts, Artefacts, Contrast-enhanced digital mammography (CEDM), Contrast-enhanced spectral mammography (CESM)

## Abstract

Contrast-enhanced digital mammography (CEDM) is a diagnostic tool for breast cancer detection. Artefacts are observed in about 10% of CEDM examinations. Understanding CEDM artefacts is important to prevent diagnostic misinterpretation. In this article, we have described the artefacts that we have commonly encountered in clinical practice; we hope to ease the recognition and help troubleshoot solutions to prevent or minimise them.

## Teaching points

- CEDM as a diagnostic tool for breast tumour evaluation.
- Identify the various CEDM artefacts
- Describe the causes of CEDM artefacts
- Discuss the assessment and possible rectification of these artefacts.

## Introduction

Contrast-enhanced digital mammography (CEDM) is an evolution of the full-field digital mammography (FFDM); it uses a dual-energy acquisition after the administration of an intravenous (IV) contrast media to assess the physiological properties of breast cancer [1]. The Food and Drug Administration approved the use of CEDM in clinical practice since 2011 [2].

CEDM acquires rapid sequential high-energy (HE) and low-energy (LE) images in craniocaudal (CC) and mediolateral oblique (MLO) projections. It has been reported that the LE images have morphological details similar to FFDM images [3] while the HE images highlight areas of contrast absorption but are not interpretable. The two images are then combined to enhance areas of contrast uptake, while cancelling anatomic noise, producing a resultant recombined image. For image interpretation, the radiologist evaluates the LE and recombined images.

At present, CEDM is indicated for the assessment of breast symptoms, work-up of inconclusive findings on mammography, staging of newly diagnosed breast cancer, evaluation of response to neoadjuvant chemotherapy and recently as an alternative to MRI in high risk screening [4].

An artefact is any error in the representation of information, introduced by patients, equipment or techniques involved. In literature, there is limited information about artefacts in CEDM [5, 6].

Like any other imaging technique, CEDM images may present artefacts. It is necessary for radiologists to be familiar with these findings to avoid diagnostic errors. All the images in this article have been acquired with a Selenia Dimensions mammography system (Hologic Marlborough, Massachusetts), which has the capability of performing FFDM, tomosynthesis and CEDM examinations. In our department, we started performing CEDM studies since 2016 and have completed more than 1300 studies. A summary of the main artefacts analysed in this article have been summarised in Table 1.

**Table 1:** Summary table of artefacts

Type of artefact	Artefact appearance	Methods to rectify
Air trapping	In recombined images, it appears as black lines.	It can be reduced by applying adequate compression between the skin and the detector or compression paddle.
Antiperspirants	In LE images, they appear as small white dots, while in recombined images, they appear as black dots.	Ask the patient to clean the breast and axillary region before the examination.
Breast implant artefact	In recombined images, breast implants produce significant artefacts which compromise the CEDM image quality.	MRI is the preferred imaging modality for patents with breast implants.
Contrast splatter	Contrast splatter appears as white dots in recombined images.	The staff who administers the contrast should not position the patient in the mammography unit, disconnect the injector tubing away from the mammography unit, clean the detector between patients.
Ghosting artefact	In recombined images, a latent image from a prior exposure is superimposed on a newly acquired image.	Recalibration of the machine can rectify this artefact.
Hair artefacts	Hair within the image are visible as thin curvilinear non-enhancing opacities.	Ensure the patient’s hair is pulled back and tied. Remove any earrings or accessories before the examination.
Halo artefact	This artefact is characterised by a curvilinear area of increased density along the edge within the recombined image. It can mask a lesion.	This artefact is not seen in the newer systems.
Misregistration artefacts	In recombined images, alternating bright and dark lines, illustrating a “zebra artefact” are seen on surgical clips.	This artefact can be decreased by reducing patient’s motion during image acquisition.
Motion artefacts	Lesions and post-biopsy markers are poorly defined.	It is reduced by adequate compression and instruct the patient to remain still.
Negative contrast enhancement	In recombined images, cysts and calcifications appear darker than the surrounding background.	This is not a true artefact and it cannot be eliminated. However, it does not compromise the image quality.
Post biopsy markers	In recombined images, markers usually appear as high attenuation structures while some are surrounded by a dark halo.	Manufacturers are developing algorithms to reduce these artefacts.
Ripple artefact	This artefact is characterised by thin black and white parallel lines.	Reducing patient anxiety might reduce this artefact.
Skin-line enhancement	In recombined images, the skin is seen as a thin rim enhancement also known as a skyline artefact.	This artefact cannot be eliminated. To verify that it is not due to a pathological thickening of the skin, check the skin thickness in LE images.
The enhancement of skin lesions	Skin angiomas may show enhancement in recombined images.	Careful clinical assessment of the skin.
Transient retention of contrast in blood vessels	In recombined images, there is a bolus of contrast seen in the blood vessels.	It is a temporary phenomenon that disappears in the subsequent acquisitions.

## Artefacts

Artefacts in CEDM can be classified as “specific”, i.e. visible only in CEDM and not visible with other imaging techniques, and “non-specific”, i.e. artefacts that can also be seen with other imaging techniques, such as mammography.

Specific CEDM artefacts are grouped together in the category called “CEDM-related factors”.

Instead, non-specific CEDM artefacts have been further categorised into artefacts visible in both CEDM and FFDM images, artefacts related to contrast media administration and quality control artefacts.

### Artefacts visible in both CEDM and FFDM images

Based on previous literature, it has already been validated that the LE images are comparable to FFDM images [3, 7]. Therefore, some artefacts that are seen in FFDM can also be visualised in CEDM images. We highlight the artefacts that are common to CEDM such as patient motion, patient-related artefacts such as hair overlying the image, antiperspirant substances and air trapping.

#### Motion artefacts

Initially, contrast mammography used a technique called “temporal subtraction”. This technique acquires two images: one before and one after the administration of contrast media. Subtraction between these two images highlights the areas of increased uptake. However, even minimal patient motion between the two acquisitions resulted in artefacts [1].

**Fig. 1:**
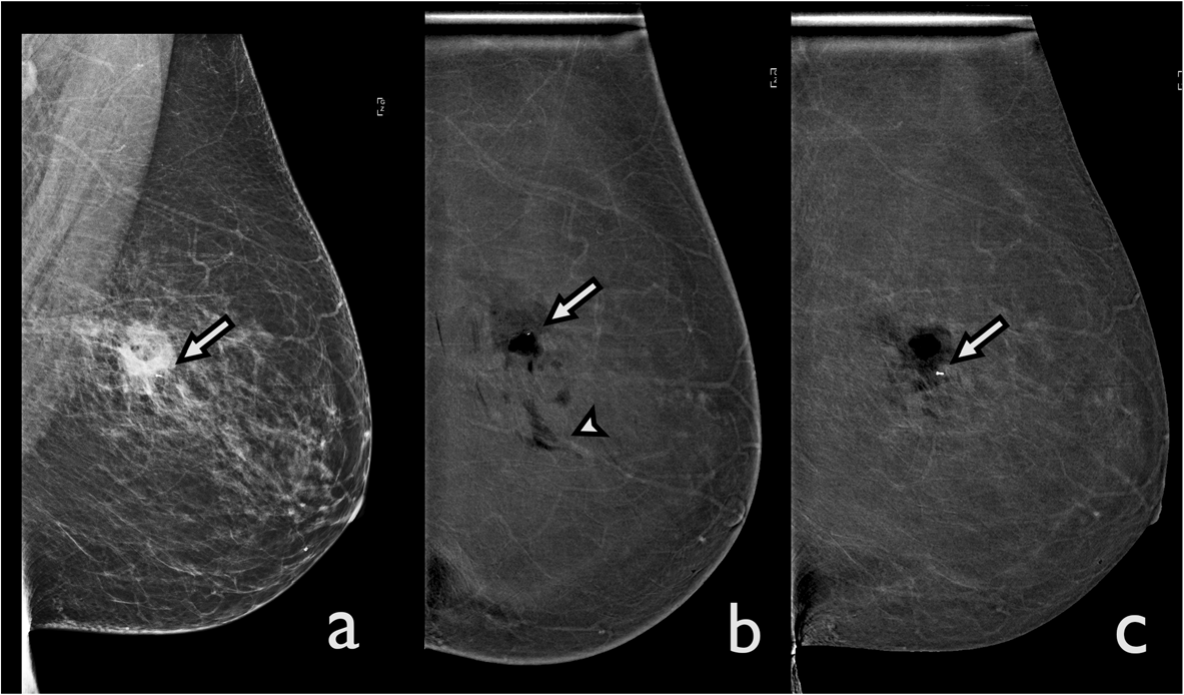
Motion artefact. A ductal carcinoma in situ was diagnosed in the left breast after an ultrasound-guided core needle biopsy. CEDM was performed for preoperative staging. **a** The LE image shows a post-biopsy hematoma with a marker (arrow). **b** The recombined CEDM image demonstrates that the margins of the hematoma are blurred secondary to motion artefacts. Some black bands are visible (arrowhead), and the post-biopsy marker appears to be in a different position from the previous image (arrow). **c** A new recombined CEDM image was performed. Here, the motion artefacts are no longer visible, and the marker appears to be in the right position (arrow)

The current CEDM process, which involves a dual energy acquisition, was developed to overcome this limitation. It is characterised by two images acquired in rapid succession by applying X-rays of distinct energy, both after the administration of contrast media. Motion artefacts have been significantly minimised due to the brief interval of time between the two exposures. However, CEDM images are still more prone to motion artefacts compared to traditional mammography because dual-energy acquisition requires longer exposure and compression times. In the recombined images, motion artefacts appear as blurring of the margins of lesions (Fig. 1). Adequate compression can reduce motion artefacts both in FFDM and CEDM [8].

#### Hair artefacts

**Fig. 2:**
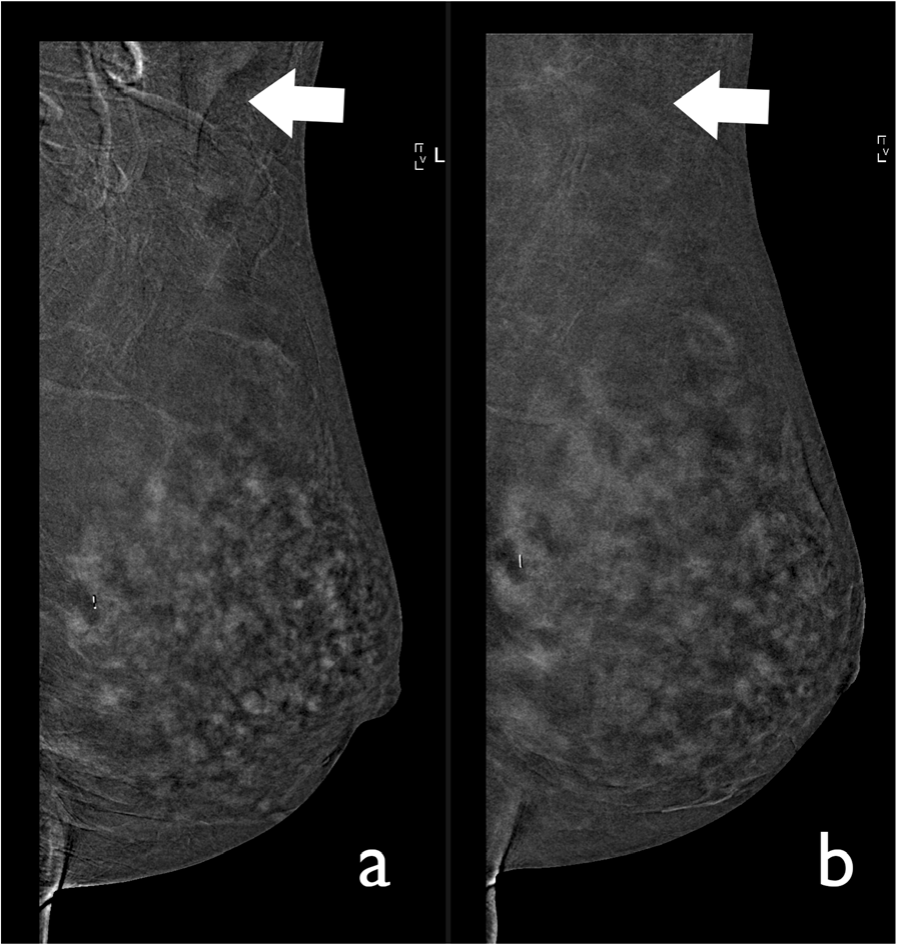
Hair artefact. CEDM was performed for preoperative staging in a 51-year-old patient. **a** The recombined image shows several curvilinear lines related to the patient’s hair (arrow). **b** Thus, we asked the patient to tie her hair before performing another acquisition. The artefact is no longer visible (arrow)

Jewellery, clothing and hair located in the field of view of the CEDM image can result in artefacts and obscure potential pathological findings (Fig. 2). Before carrying out the examination, it is important to remove any clothes and accessories that may be projected in the image and ensure the patients hair is pulled back and tied [9].

#### Antiperspirant artefacts

**Fig. 3:**
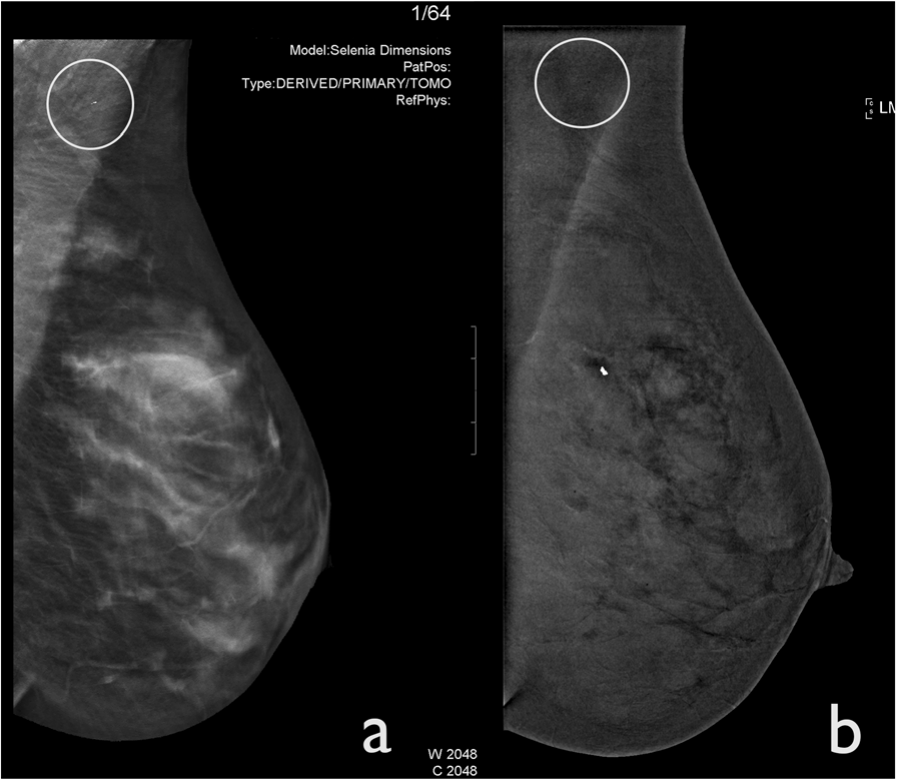
Antiperspirant artefact. CEDM was performed for staging an IDC in the right breast of a 59-year-old woman. **a** Left MLO tomosynthesis shows a little white spot in the axillary region caused by the presence of the antiperspirant substance (circle). **b** The CEDM-recombined image demonstrates that antiperspirant is barely perceptible as a small, black dot (circle)

The active ingredients of antiperspirants are complex-aluminium-based; these substances produce radiopaque particles that can mimic microcalcifications in LE images. Usually, antiperspirant particles appear black on CEDM recombined images (Fig. 3). It is important for technologists to recognise this artefact and to ask the patient to clean the axilla or skin folds before the subsequent image acquisition is performed.

#### Air trapping artefacts

**Fig. 4:**
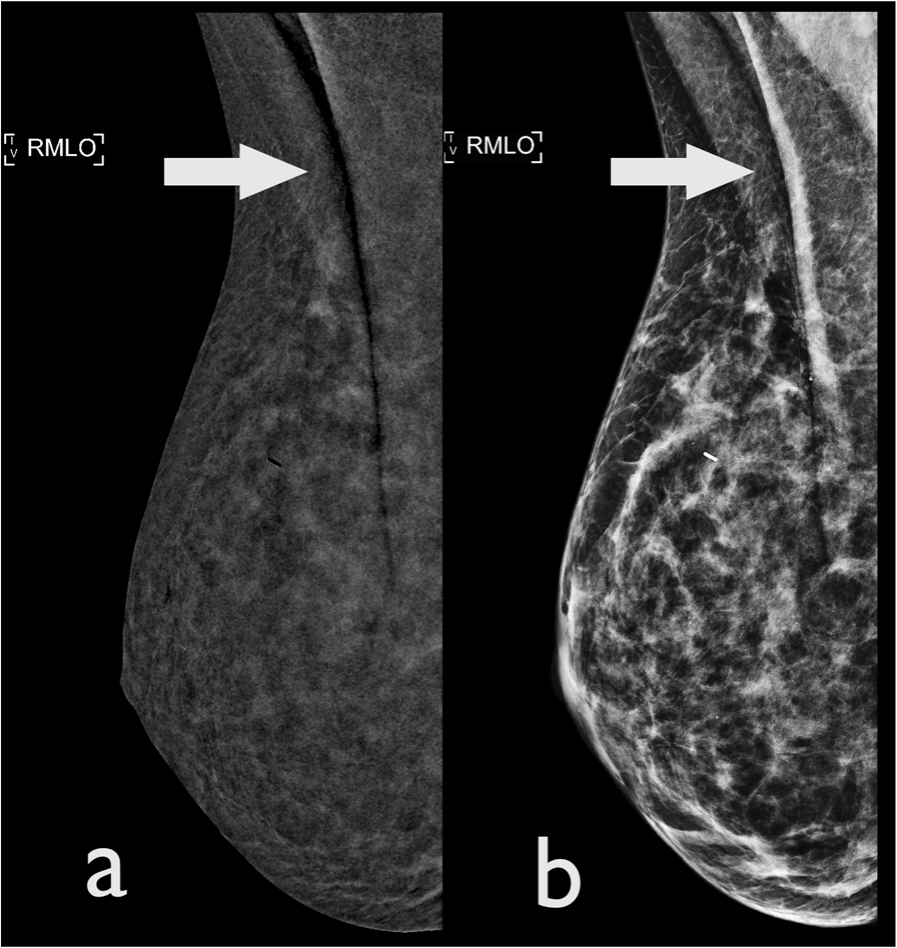
Air trapping artefacts: A CEDM performed for problem solving in a 52-year-old woman, using a (**a**) CEDM-recombined image and (**b**) LE image. Both images demonstrate the presence of a vertical, black band (arrows) secondary to trapped air

Air trapping is a common artefact caused by partial contact between the skin and the detector or compression paddle [1]. This leads to the presence of air, which creates a dark artefact in the configuration of the area of incomplete contact, possibly hiding underlying abnormalities (Fig. 4).

### Contrast-related factors

Several contrast-related factors can affect the image quality in CEDM.

#### Contrast splatter

**Fig. 5:**
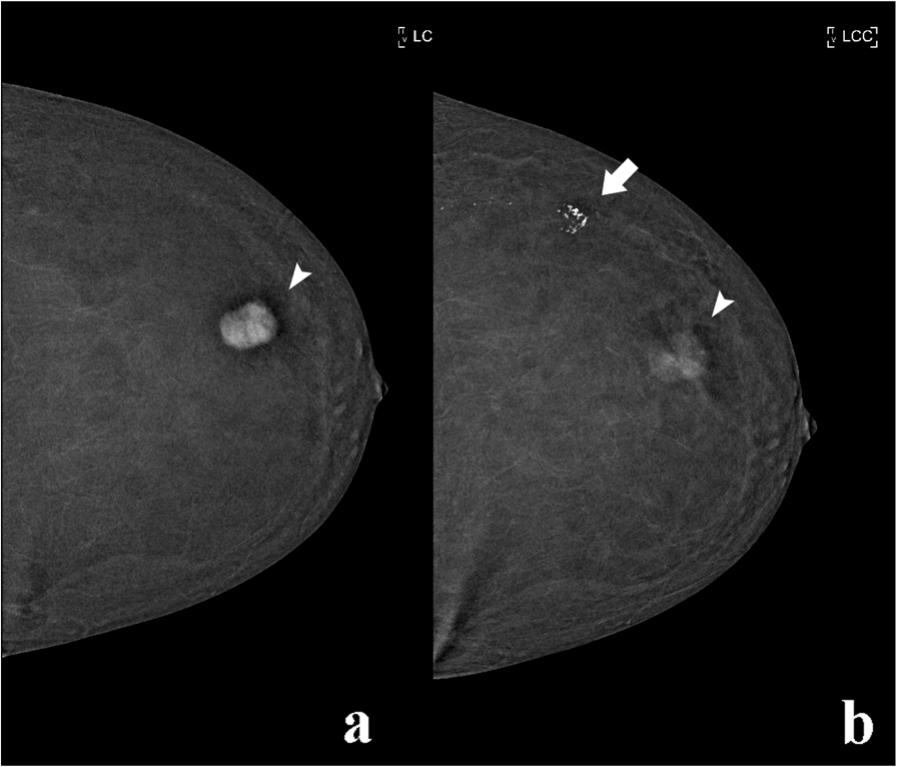
Contrast splatter. CEDM was performed in a 55-year-old woman for preoperative staging. **a** The recombined image shows an avidly enhancing lesion in the outer quadrant (arrowhead). **b** A delayed acquisition was performed where contrast contamination of the skin is visible (arrow). Contrast washout is also demonstrated within the lesion (arrowhead)

It is critical to pay close attention to the technique during contrast administration to prevent contrast contamination. When detaching the tubing from the power injector, the contrast media may accidentally splatter onto the adjacent equipment or skin, resulting in the appearance of small white dots on recombined images, which may be confused with microcalcifications (Fig. 5).

However, it should be noted that while calcifications are white on FFDM and LE CEDM images, they appear black on CEDM recombined images.

To avoid this artefact from occurring, nurses or doctors must administer contrast media away from the mammography unit. Preferably, the staff that administers the contrast media should not assist in patient positioning [10]. Carefully cleaning the region to be examined and the detector of the mammography unit prior to subsequent acquisitions can resolve this artefact.

#### Transient retention of contrast in blood vessels

**Fig. 6:**
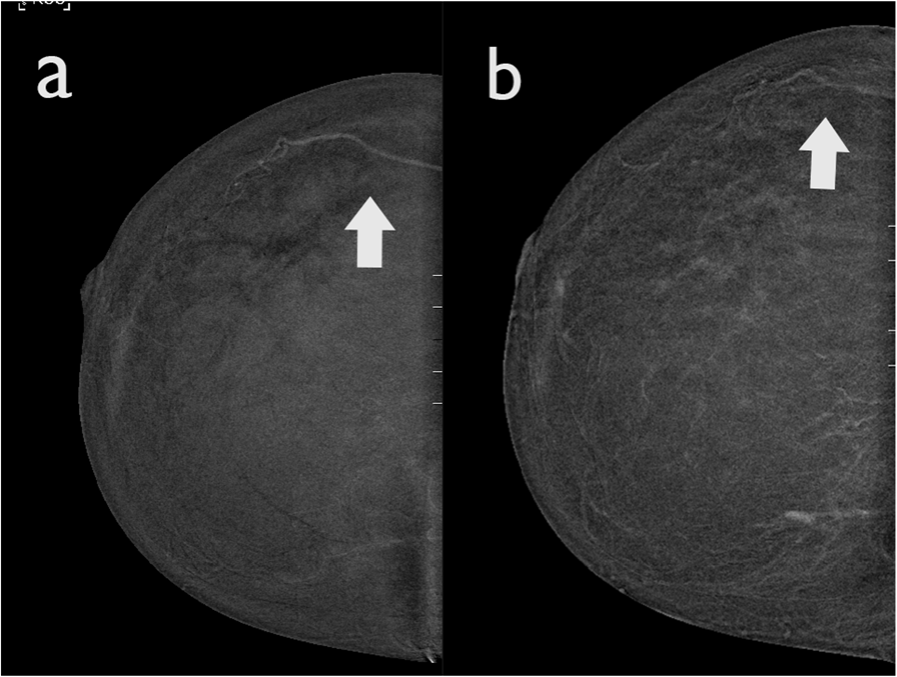
Transient retention of contrast in blood vessels: a CEDM was performed in a 66-year-old woman to evaluate a suspicious finding in the left breast on mammography. **a** The right, recombined image shows a bolus of contrast within a blood vessel (arrows); **b** this artefact disappeared is less promiscuous in the following CEDM acquisition (arrow)

The retention of contrast media in the blood vessels is frequent. However, it does not compromise image quality (Fig. 6). This phenomenon is typically due to premature breast compression and it is not visualised in the subsequent image acquisition of the same breast.

### CEDM-related factors

This category includes artefacts that are unique to CEDM.

#### Post biopsy markers

**Fig. 7:**
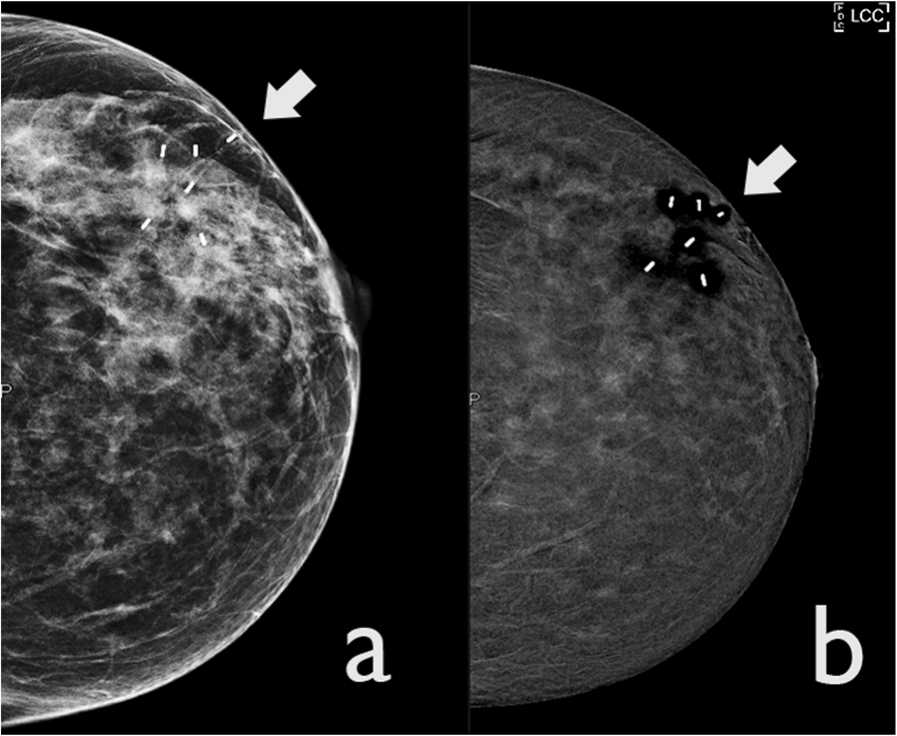
Artefact due to the presence of post ultrasound guided biopsy markers: **a** LE CEDM image. There are some markers seen at the outer quadrant of the left breast (arrow); **b** in the recombined image, the markers are seen as high attenuation structures and they demonstrate a surrounding dark halo

Placing markers after breast biopsy is very common. Markers of different composition and materials can demonstrate a variable appearance. In our centre, titanium markers are inserted in case of vacuum assisted biopsy while zirconium-oxide markers are inserted during ultrasound-guided biopsy procedures. On the recombined CEDM images, all markers appear as high attenuation structures; however, a surrounding dark halo is occasionally seen with the zirconium oxide markers (Fig. 7).

#### Breast implant artefacts

**Fig. 8:**
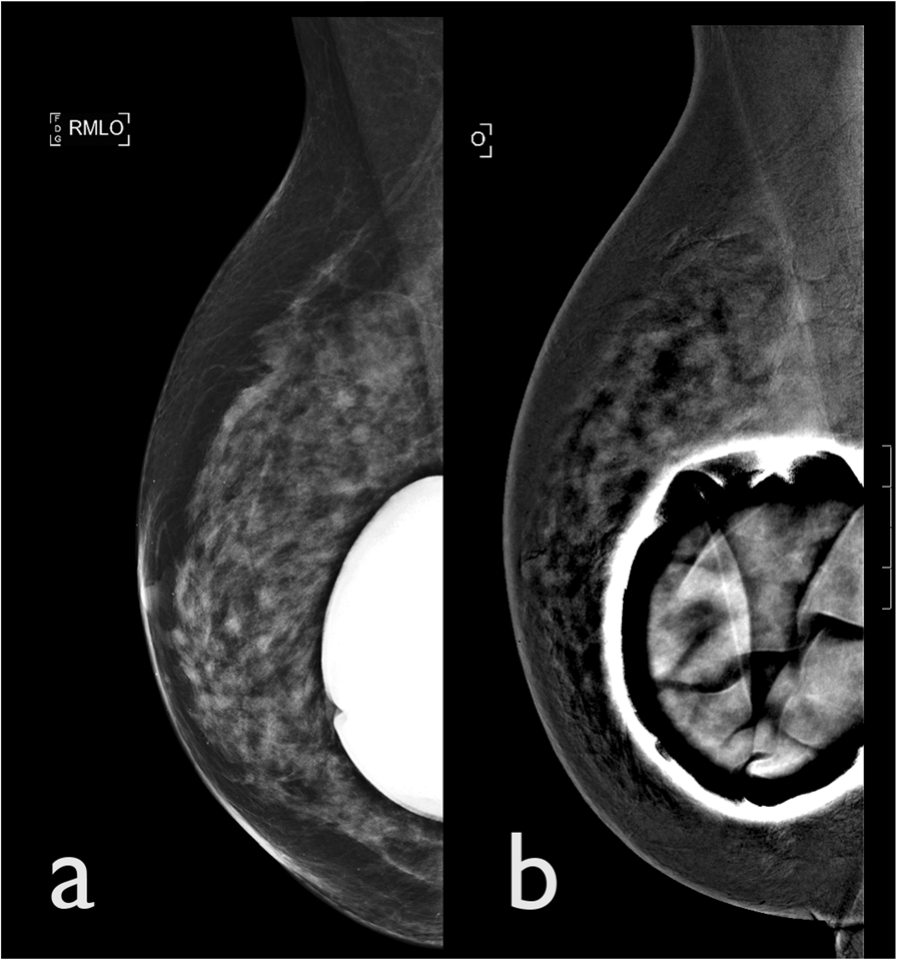
Breast implant artefact. A CEDM was performed for suspected microcalcifications in a 55-year-old woman with a right breast implant. **a** LE CEDM image shows a breast implant in situ. **b** CEDM recombined image presents a significant post-processing artefacts secondary to the presence of the implants

Breast implant rupture can lead to either intracapsular or extracapsular involvement, which requires them to be removed and replaced. The sensitivity of MRI in assessing the integrity of breast implants is about 90% [11]. Breast implants tend to produce significant artefacts on CEDM recombined images, which may obscure an underlying pathology (Fig. 8). Therefore, MRI continues to be the imaging modality of choice in women with breast implants.

#### Negative contrast enhancement

**Fig. 9:**
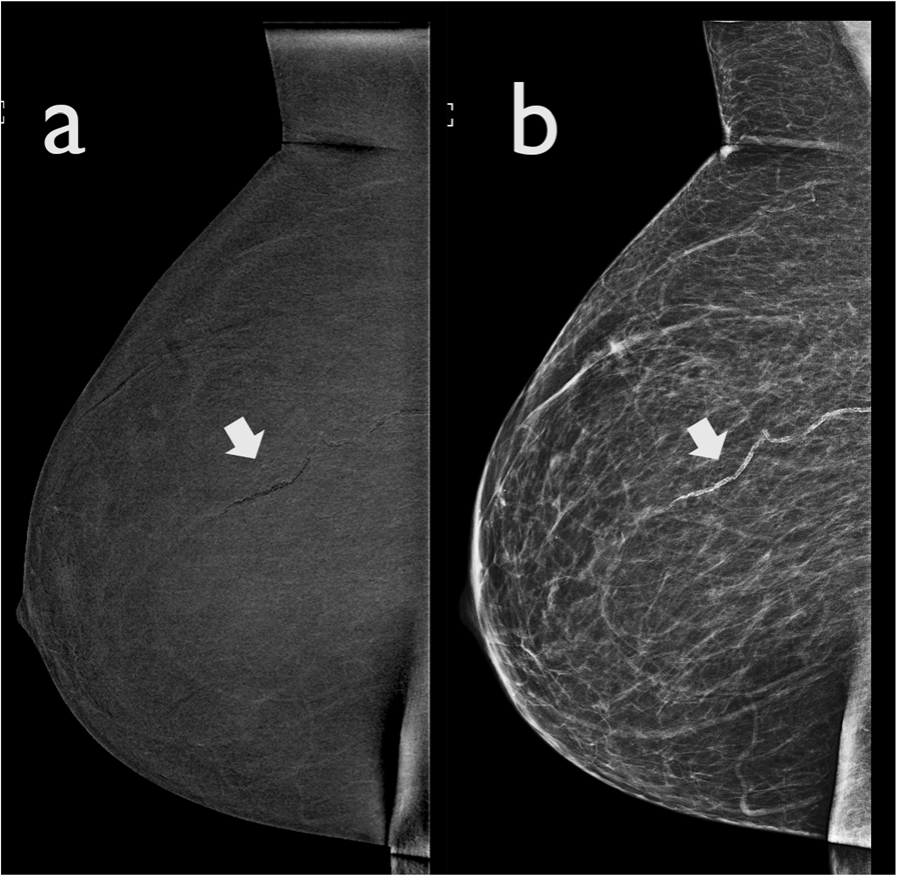
Negative contrast enhancement. **a** CEDM recombined image presents an irregular, tubular structure (arrow), which is black in relation to the surrounding background. **b** In LE CEDM image, this wavy band corresponds to a calcified blood vessel (arrow)

In recombined CEDM images, cysts, large calcifications or post-biopsy hematomas demonstrate an area of rim enhancing hypodensity in relation to the surrounding background giving it a “negative contrast enhancement” appearance which is also referred to as the “eclipse sign” (Fig. 9). This phenomenon is actually not a true artefact, but is in fact a natural consequence of the acquisition technique.

#### Halo artefact

This is a software-processing artefact also known as “breast-within-a-breast artefact” [5]. It occurs due to a rapid difference of scattered radiation between different breast thicknesses separating the central and peripheral regions causing the software-processing algorithm to create a false exaggerated boundary.

**Fig. 10:**
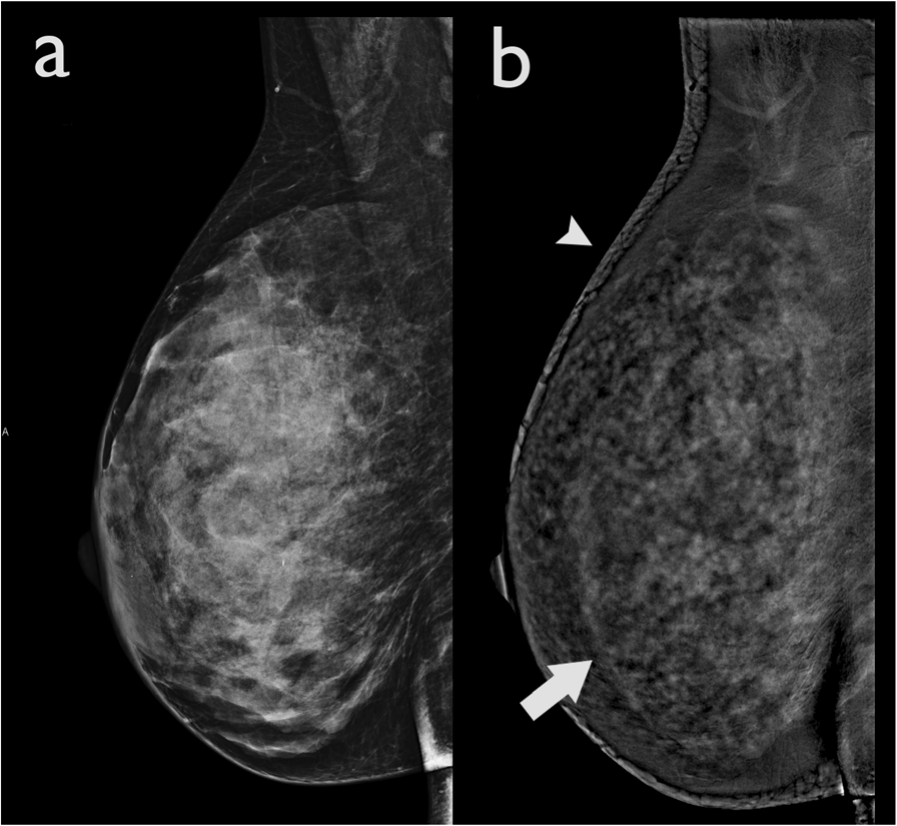
Halo artefact in a 52-year-old woman. **a** The LE CEDM image demonstrates a dense breast with no show focal alterations, and (**b**) the recombined image presents an apparent “breast-within-a-breast” artefact (arrow). The skin artefact that is observed in the right breast (arrowhead) is caused by detector saturation in the skin region due to a high detector signal

This artefact is characterised by a curved line of high density, parallel to the edge of the skin (Fig. 10). This artefact does not usually compromise image quality. However, in presence of prominent parenchymal enhancement, a small mass located parallel to this high-density artefact may be obscured. This artefact appears to be vendor specific.

#### Ripple artefact

**Fig. 11:**
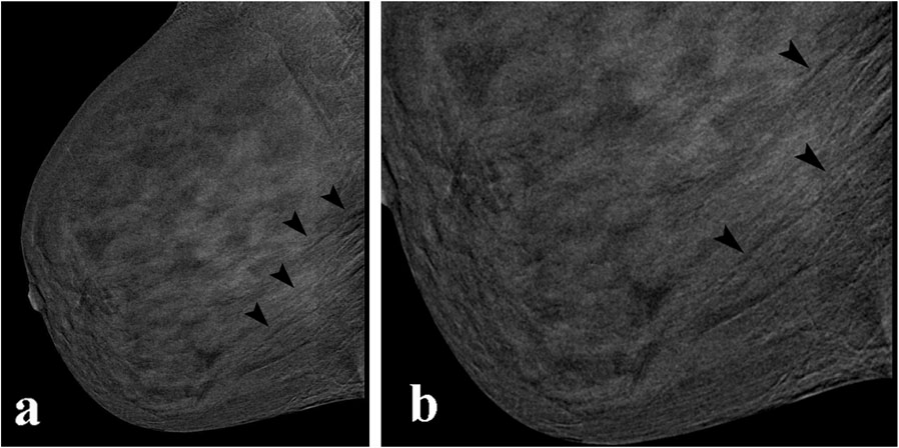
Ripple artefact. CEDM performed for preoperative staging. The arrowheads are indicating the ripple artefact, which are seen as thin alternate dark and light lines in the recombined image (**a**). The artefact is more visible in the magnified view (**b**, arrowheads)

Ripple artefacts are very commonly visible on recombined images as alternating black and white lines. They are mainly seen in the MLO projections (Fig. 11). Dromain et al. [12] suggested that this may be attributed to patient motion, due to the short interval between the low- and high-energy exposures. However, there are some authors [5] who attribute it to cardiac pulsations transmitted through the chest wall as it more commonly seen in the lower quadrants of MLO projections of the left breast. Reducing patient anxiety might reduce this artefact; however, it does not compromise the image quality.

#### Misregistration artefacts

**Fig. 12:**
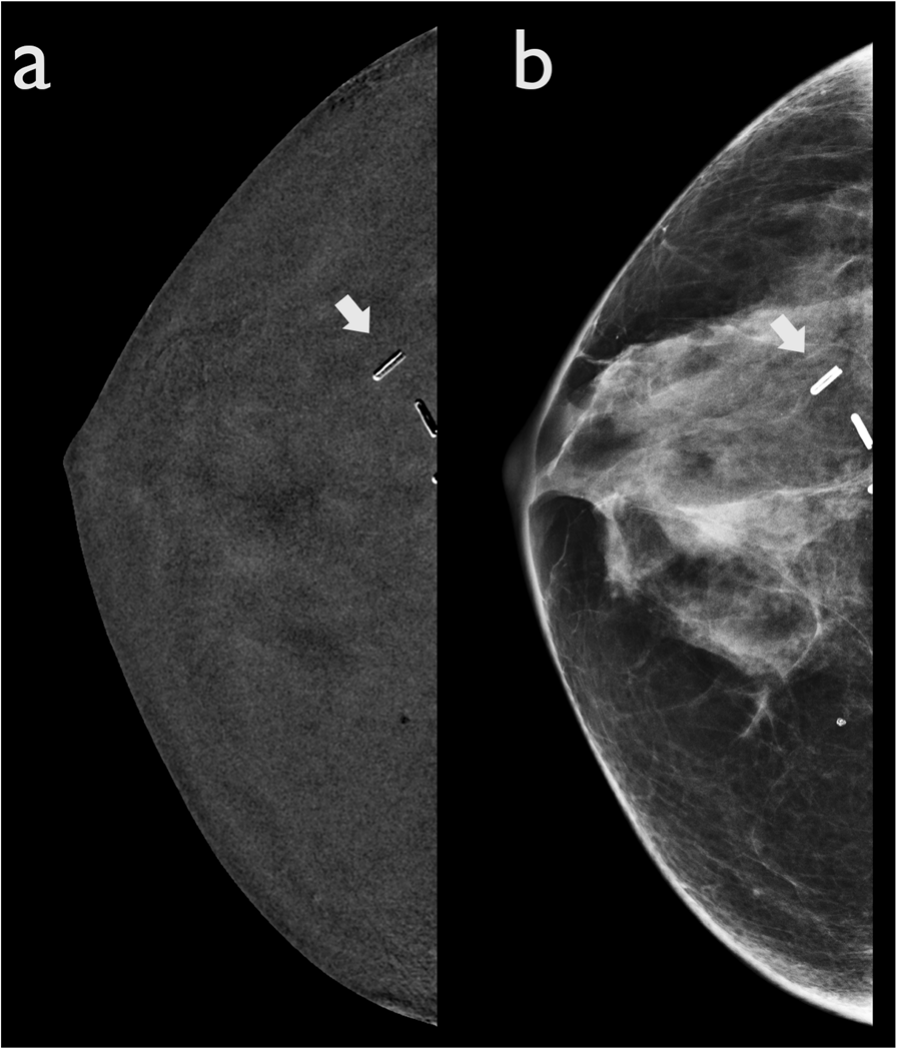
Misregistration artefact. A woman with a history of previous right quadrantectomy. **a** CEDM recombined image shows post-surgical clips with alternating bright and dark lines (arrow). **b** The same clips are also visible in LE CEDM images (arrow)

Misregistration artefacts are commonly observed in relation to surgical clips, vessels and calcifications. It is seen as alternating bright and dark lines, illustrating a “zebra artefact” seen exclusively on recombined images (Fig. 12). Even minimal motion between the LE and HE images causes misalignment of the images, resulting in imprecise subtraction.

#### Skin-line enhancement and enhancing skin lesions

**Fig. 13:**
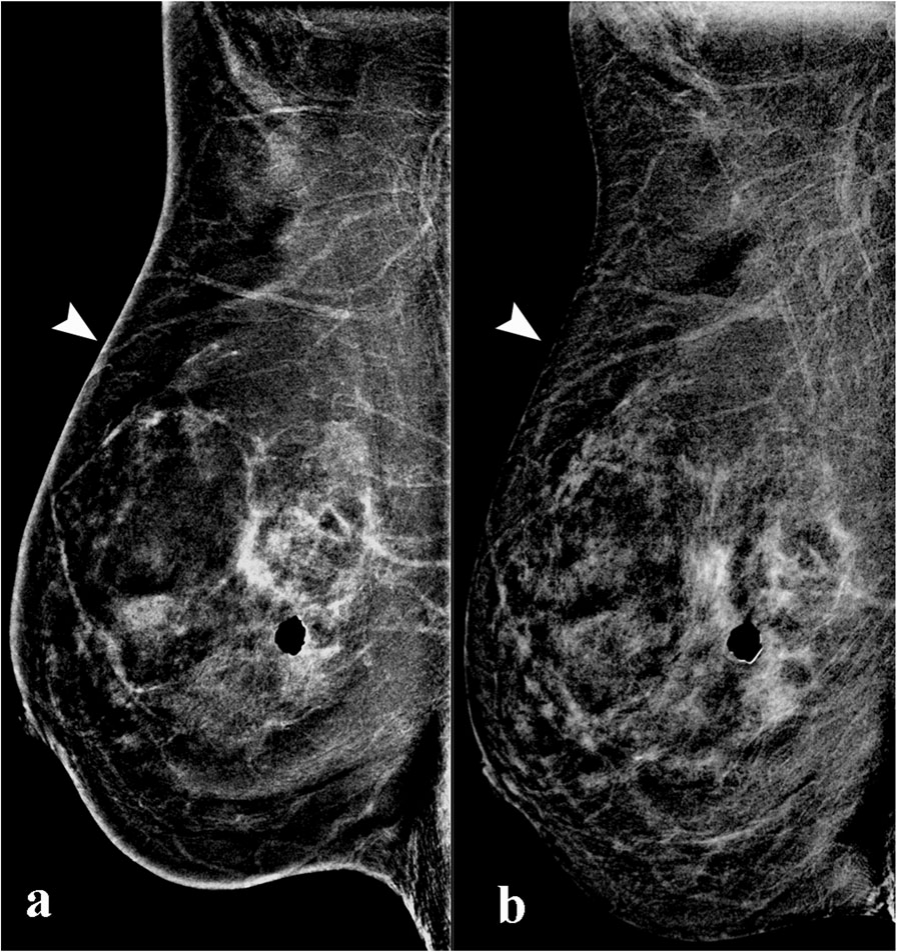
Skin-line artefact. **a** The early-recombined image demonstrates a marked enhancement of the skin line. **b** The skin enhancement is not appreciated in the delayed recombined CEDM acquisition

The skin does not usually demonstrate enhancement in recombined images; however, it may show a thin rim of enhancement. This artefact may be caused by non-uniform scatter radiation and difference in skin thickness throughout the breast. This artefact is also known as “skyline artefact” (Fig. 13). It is necessary to check the skin thickness in the LE images, to verify that the skin is not truly affected by a disease.

**Fig. 14:**
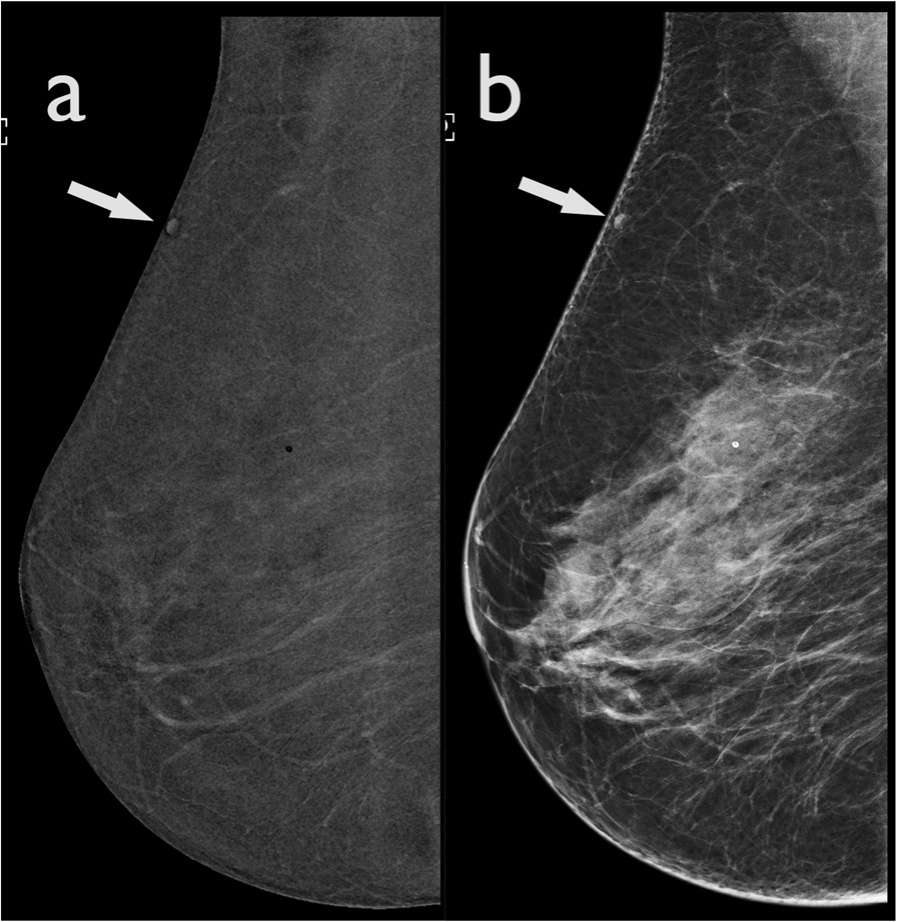
Enhancement of skin lesions. **a** The recombined image demonstrates a small oval enhancing lesion in the upper quadrant of the right breast, which could mimic a small enhancing mass (arrow). **b** The LE CEDM image shows an oval hyperdensity formation in the upper quadrant. Evaluation of the patient’s skin in this region confirmed the presence of a small skin angioma

Moreover, some vascular skin formations, such as skin angiomas, may show contrast enhancement, mimicking the presence of suspicious lesions (Fig. 14). A careful clinical examination of the patient’s skin may be necessary.

### Quality control (QC) artefacts

These artefacts can severely compromise the image quality. It is important to train all technologists and schedule specific times for the daily QC processes and specific days for the weekly QC processes. It is a good practice to test the system on a phantom prior to the CEDM imaging schedule.

#### Ghosting artefact

**Fig. 15:**
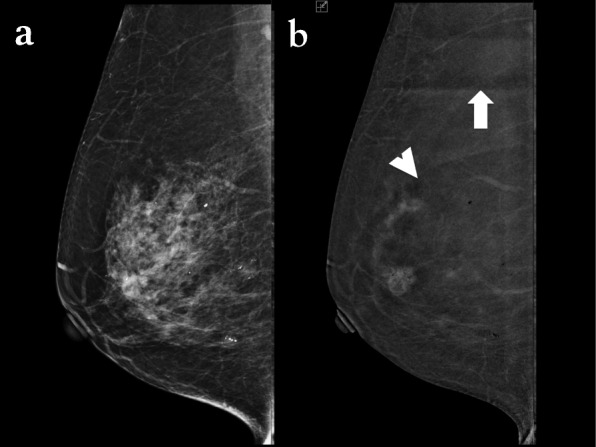
Ghosting artefact: a 50-year-old woman presented with a right breast lump. **a** The LE CEDM image shows no obvious artefacts, and (**b**) ghosting of the latent CC view (arrowhead) of the previously imaged breast is projected within the MLO image and an axillary line artefact (arrow) is also evident in this image

This artefact occurs when a previous image acquisition remains latent and overlaps with the subsequent image. In CEDM, rapid transitions between acquisitions can be the cause of a persistent latent signal that overlaps the new acquisition (Fig. 15). This artefact is not commonly seen and recalibrating the machine to remove the memory of the previous image can rectify this complication.

## Conclusion

Image artefacts are common on CESM examinations and it is important to recognise them so that radiologists can differentiate abnormal enhancement from artefacts.

It is important that the technologist, radiologist and physicist become familiar and investigate the CEDM artefacts further as and when they occur, to optimise the image quality and avoid interpretive pitfalls.

## Data Availability

Not applicable

## Abbreviations

CC: Craniocaudal
CEDM: Contrast-enhanced digital mammography
FFDM: Full-field digital mammography
HE: High-energy
LE: Low-energy
MLO: Mediolateral oblique
QC: Quality control
